# The Neuroprotective Effect of Rapamycin as a Modulator of the mTOR-NF-κB Axis during Retinal Inflammation

**DOI:** 10.1371/journal.pone.0146517

**Published:** 2016-01-15

**Authors:** Tomohiro Okamoto, Yoko Ozawa, Mamoru Kamoshita, Hideto Osada, Eriko Toda, Toshihide Kurihara, Norihiro Nagai, Kazuo Umezawa, Kazuo Tsubota

**Affiliations:** 1 Laboratory of Retinal Cell Biology, Department of Ophthalmology, Keio University School of Medicine, 35 Shinanomachi, Shinjuku-ku, Tokyo, 160–8582, Japan; 2 Department of Ophthalmology, Keio University School of Medicine, 35 Shinanomachi, Shinjuku-ku, Tokyo, 160–8582, Japan; 3 Department of Molecular Target Medicine Screening, Aichi Medical University School of Medicine, Nagakute, Aichi, 480–1195, Japan; Dalhousie University, CANADA

## Abstract

**Purpose:**

The determination of the molecular mechanism underlying retinal pathogenesis and visual dysfunction during innate inflammation, and the treatment effect of rapamycin thereon.

**Methods:**

The endotoxin-induced uveitis and retinitis mouse model was established by injecting lipopolysaccharide. The mice were subsequently treated with rapamycin, a mammalian target of rapamycin (mTOR) inhibitor. The rhodopsin mRNA and protein expression level in the retina and the photoreceptor outer segment (OS) length in immunohistochemical stainings were measured, and visual function was recorded by electroretinography. Inflammatory cytokines, their related molecules, mTOR, and LC3 levels were measured by real-time PCR and/or immunoblotting. Leukocyte adhesion during inflammation was analyzed using concanavalin A lectin.

**Results:**

The post-transcriptional reduction in the visual pigment of rod photoreceptor cells, rhodopsin, OS shortening, and rod photoreceptor cell dysfunction during inflammation were suppressed by rapamycin. Activation of nuclear factor kappa-light-chain-enhancer of activated B cells (NF-κB) and induction of inflammatory cytokines, such as interleukin-6 (IL-6) and monocyte chemoattractant protein-1 (MCP-1), and the activation of the downstream signaling protein, signal transducer and activator of transcription 3 (STAT3), which reduces rhodopsin in the retina during inflammation, were attenuated by rapamycin. Increased leukocyte adhesion was also attenuated by rapamycin. Interestingly, although mTOR activation was observed after NF-κB activation, mTOR inhibition suppressed NF-κB activation at the early phase, indicating that the basal level of activated mTOR was sufficient to activate NF-κB in the retina. In addition, the inhibition of NF-κB suppressed mTOR activation, suggesting a positive feedback loop of mTOR and NF-κB during inflammation. The ratio of LC3II to LC3I, which reflects autophagy induction, was not changed by inflammation but was increased by rapamycin.

**Conclusions:**

Our results propose the potential use of rapamycin as a neuroprotective therapy to suppress local activated mTOR levels, related inflammatory molecules, and the subsequent visual dysfunction during retinal inflammation.

## Introduction

Recent progress in the medical sciences has shown that inflammation partakes in the pathogenesis of several systemic diseases including arthritis [1], gastrointestinal diseases [2], arteriosclerosis [3], hypertension [4], and metabolic syndrome [5], as well as in neurodegenerative diseases, such as Alzheimer’s disease [6, 7] and diabetic retinopathy [8–11]. Therefore, more and novel anti-inflammatory therapies may contribute to improved disease prognosis in each of these disease areas [12, 13]. Research into anti-inflammatory treatments as alternatives to the currently used ones, such as steroids, has found rapamycin, a negative regulator of the mammalian target of rapamycin (mTOR) [14], as one of the promising drugs. Indeed, rapamycin is currently in global clinical trials for the treatment of the non-infectious ocular inflammatory disease, uveitis [15], and is already a clinically available immunosuppressant [16]. However, the molecular mechanisms underlying rapamycin’s treatment effects are not fully understood.

mTOR is an atypical serine/threonine protein kinase that acts as the phosphoinositide 3-kinase (PI3K)-related kinase family, forming two distinct complexes called mTOR complex 1 (mTORC1) and 2 (mTORC2). Although rapamycin is believed to be a specific inhibitor of mTORC1 [14], a recent study has shown that it can also inhibit mTORC2 [17]. While mTORC1 regulates numerous cellular processes including protein synthesis related to growth and differentiation, and plays an important role in cancer therapy, mTORC2 has a regulatory role in the insulin-signaling cascade, which may, thus, be treated by rapamycin [17].

Uncontrolled inflammation of the uvea may spread into the retina causing loss of vision. In fact, an experimental uveitis model has shown that retinal inflammation reduced the visual pigment rhodopsin and impaired visual function [18–26]. Theoretically, rapamycin might suppress T cell activation during the chronic phase of non-infectious uveitis [15], which was the original concept for the clinical trials. However, because half of the non-infectious uveitis cases are caused by inflammation of unknown etiology without the involvement of T cells, the present concept of rapamycin’s mechanism of action may limit its therapeutic application. Indeed, the pathogenesis of most non-infectious uveitis cases and the subsequent retinitis primarily involves innate inflammation. Thus, evaluating the treatment effects of rapamycin may aid in defining more appropriate applications of rapamycin; if rapamycin can reduce innate inflammation, its therapeutic application may be expanded to non-T-cell mediated uveitis.

Herein, the neuroprotective effect of rapamycin and its underlying molecular mechanisms were evaluated and partly demonstrated using the endotoxin-induced uveitis and retinitis (EIU) mouse model of innate inflammation.

## Materials and Methods

### Ethics Statement

All animal experiments were conducted in accordance with the Association for Research in Vision and Ophthalmology (ARVO) Statement for the Use of Animals in Ophthalmic and Vision Research, the ARRIVE (Animal Research: Reporting In Vivo Experiments) guidelines, and the guideline for the ethics committee of Keio University, Tokyo, Japan. The protocol for this study was approved by the Keio University Institutional Animal Care and Use Committee (permission no. 08002) (Tokyo, Japan).

### Animals and treatments

Six-week-old male C57BL/6 mice were purchased (Clea Japan, Tokyo, Japan) and maintained at 22 ± 2°C in an air-conditioned room under a 12-h dark/light cycle (lights on from 0800 to 2000), with access to a standard diet (Clea Japan) and tap water ad libitum. The mice were housed five per cage, and their physiological condition was checked several times a week before lipopolysaccharide (LPS) injection, and every day after LPS injection. An euthanasia protocol based on pentobarbital sodium was incorporated in the study design for seriously ill mice to minimize pain and distress; however, no seriously ill or dead mice were found before the study endpoint. The mice received a single intraperitoneal injection of 6.0 mg/kg body weight (BW) LPS from *Escherichia coli* (Sigma-Aldrich, St. Louis, MO, USA) in phosphate-buffered saline (PBS) to establish the EIU model. The control mice received a single intraperitoneal injection of PBS. One hour after the LPS or PBS injection, the mice were treated either with the specific mTOR inhibitor rapamycin (dissolved in dimethyl sulfoxide [DMSO, 25 mg/mL] and stored at −20°C; Selleck Chemicals, Houston, TX, USA) at 6.0 mg/kg BW, or with PBS containing 4% DMSO as the vehicle. The stock solution was diluted with 5% polyethylene glycol 400 and 5% Tween 80 immediately before the intraperitoneal injections. Alternatively, 2 hours before the LPS injection, the mice were intraperitoneally injected either with the specific NF-κB inhibitor dehydroxymethylepoxyquinomicin (DHMEQ, provided by Dr. Kazuo Umezawa, Aichi Medical University, Aichi, Japan), which inhibits the nuclear translocation of NF-κB and its subsequent DNA binding, at 10 mg/kg BW, or with PBS containing 4% DMSO as the vehicle [26]. For sampling of the retina, the mice were anesthetized with an intraperitoneal injection of pentobarbital sodium (Dainippon Sumitomo Pharmaceutical Co., Osaka, Japan) at 60 mg/kg BW and were sacrificed by cervical dislocation.

### Immunoblot Analyses

The eyes of the mice were enucleated, and each retina was isolated and placed in lysis buffer containing a protease inhibitor cocktail (Complete, EDTA-free; Roche, Mannheim, Germany) and phosphatase inhibitor cocktails 2 and 3 (Sigma-Aldrich, St. Louis, MO, USA). The resultant lysate was treated with Laemmli’s sample buffer and was separated by 10% SDS-polyacrylamide gel electrophoresis. The proteins were electrically transferred to a polyvinylidene fluoride membrane (Immobilon-P; Millipore, Bedford, MA, USA) in a Trans-Blot SD Cell (Bio-Rad Laboratories, Hercules, CA, USA). After the transfer, the membrane was blocked with 5% skim milk in TBS-T or TNB blocking buffer (0.1 M Tris-HCl, pH 7.5, 0.15 M NaCl, 0.5% TSA Blocking Reagent [PerkinElmer Life Sciences, Waltham, MA, USA]), and was subsequently incubated overnight at 4°C with a rabbit anti-rhodopsin (1:100,000; LSL, Osaka, Japan), rabbit anti-phospho-nuclear factor kappa-light-chain-enhancer of activated B cells (NF-κB) p65 (Ser536) (93H1, 1:1,000), rabbit anti-phospho-signal transducer and activator of transcription 3 (STAT3, 1:1,000), or rabbit anti-phospho-mTOR (1:1,000) antibody (all purchased from Cell Signaling Technology, Beverly MA, USA), or LC3 antibody (1:1,000; Novus Biologicals, Littleton, CO, USA), and either with a mouse anti-α-tubulin (1:1,000; Cell Signaling Technology, Beverly MA, USA) or mouse anti-glyceraldehyde 3-phosphate dehydrogenase (GAPDH, 1:100,000; Cell Signaling Technology, Beverly MA, USA) antibody. The membrane was subsequently incubated with the appropriate horseradish peroxidase-conjugated secondary antibody. Finally, the protein signals were detected using the enhanced chemiluminescence system (ECL Blotting Analysis System; Amersham, Arlington Heights, IL, USA), and were measured and normalized to those of α-tubulin or GAPDH with the ImageJ program (developed by Wayne Rasband, National Institutes of Health, Bethesda, MD, USA; available at http://rsb.info.nih.gov/ij/index.html).

### Real-Time RT-PCR

Total RNA was isolated from the retina with TRIzole (Life technology, Carlsbad, CA, USA) and was reverse-transcribed using the High Capacity RNA-to-cDNA Master Mix (Applied Biosystems, Foster City, CA, USA), according to the manufacturer’s instructions. The *rhodopsin* mRNA levels were normalized to those of *GAPDH*. The following forward and reverse primer sequences were used: *rhodopsin* forward 5′-AACTTCGGCCCCATCTTCA-3′ and *rhodopsin* reverse 5′-CAGTGGATTCTTGCCGCAG-3′, *GAPDH* forward 5′-AACTTCGGCCCCATCTTCA-3′ and *GAPDH* reverse 5′-GATGACCCTTTTGGCTCCAC-3′, and *interleukin 6* (*IL-6*) forward 5′-AAGTCGGAGGCTTAATTACACATGT-3′ and *IL-*6 reverse 5′-CCATTGCACAACTCTTTTCTCATTC-3′. For monocyte chemoattractant protein-1 (*MCP-1*), TaqMan Gene Expression Assay for the specific gene (TaqMan probe assay ID; Mm00441242_m1 Life Technologies) was used. Real-time PCR was performed using the StepOnePlus™ PCR system (Applied Biosystems, Foster City, CA, USA), and the gene expression was quantified using the delta delta CT method.

### Immunohistochemistry

Retinal sections (10-μm–thick) were fixed in 4% paraformaldehyde. The sections were incubated with a rabbit anti-rhodopsin antibody (1:10,000; LSL) followed by incubation with Alexa 488-conjugated goat anti-rabbit IgG. Nuclei were counterstained with 2 μg/mL Cellstain-DAPI solution (Dojindo Molecular Technologies, Kumamoto, Japan). Outer segment (OS) lengths were measured in the posterior retina at four points that were at a distance between 200 and 500 μm from the optic nerve, two on either side, using the ImageJ program, and the lengths were averaged. All sections were examined under a microscope equipped with a digital camera (Olympus Co., Tokyo, Japan).

### Electroretinography

The mice were dark-adapted for at least 12 hours and were prepared under dim red illumination. They were anesthetized with 60 mg/kg BW of pentobarbital sodium (Dainippon Sumitomo Pharmaceutical Co., Osaka, Japan) and were kept on a heating pad throughout the experiment. The pupils were dilated with one drop of a mixture of 0.5% tropicamide and 0.5% phenylephrine (Santen Pharmaceutical Co., Osaka, Japan). The ground and reference electrode was placed on the tail and in the mouth, respectively. The active electrodes were gold wires and were placed on the cornea. The recordings were made with a PowerLab System 2/25 (AD Instruments, New South Wales, Australia). Full-field scotopic electroretinographs were measured in response to a flash at intensities ranging from −2.12 to 2.89 log cd sec/m^2^. The responses were differentially amplified and were filtered through a digital bandpass filter ranging from 0.3 to 1000 Hz. Each stimulus was delivered through a commercial stimulator (Ganzfeld System SG-2002; LKC Technologies, Inc., Gaithersburg, MD, USA). The implicit times of the a- and b-waves were measured from the onset of the stimulus to the peak of each wave. The amplitude of the a-wave was measured from the baseline to the trough of the a-wave, and the amplitude of the b-wave was measured from the trough of the a-wave to the peak of the b-wave. The peak points were automatically indicated by the system and were confirmed by the experimenter.

### Enzyme-linked immunosorbent assay

The inflammatory cytokines in the retinal tissue were measured using enzyme-linked immunosorbent assay (ELISA) kits for IL-6 and MCP-1 (R&D Systems, Minneapolis, MN, USA). All spectrophotometric readings were performed using an absorption spectrometer (Wallac ARVO SX 1420 Multilabel Counter; PerkinElmer). All procedures were performed according to the manufacturer’s instructions.

### Quantification of Retinal Adherent Leukocytes

The retinal vasculature and adherent leukocytes were imaged by perfusion-labeling with fluorescein isothiocyanate (FITC)-coupled concanavalin A lectin (ConA; Sigma-Aldrich, St. Louis, MO) as described previously [9, 20, 22, 27, 28]. Briefly, 24 hours after LPS injection, the chest cavity was opened and a 24-gauge cannula was introduced into the left ventricle under deep anesthesia. After injection of 2 mL of PBS to remove erythrocytes and nonadherent leukocytes, 2 mL of FITC-conjugated ConA was perfused. After the eyes were enucleated, the retinas were flatmounted. The flatmounts were imaged with an epifluorescence microscope (BZ-9000; Keyence, Osaka, Japan), and the total number of ConA-stained adherent leukocytes per retina was counted in a blinded manner.

### Statistical analyses

All results are expressed as the mean ± SD. One way ANOVA with Tukey’s post hoc test was used to assess the statistical significance of differences; p < 0.05 was regarded as significant.

## Results

### Rapamycin suppressed rhodopsin reduction and OS shortening during inflammation in a post-transcriptional manner

**Fig 1:**
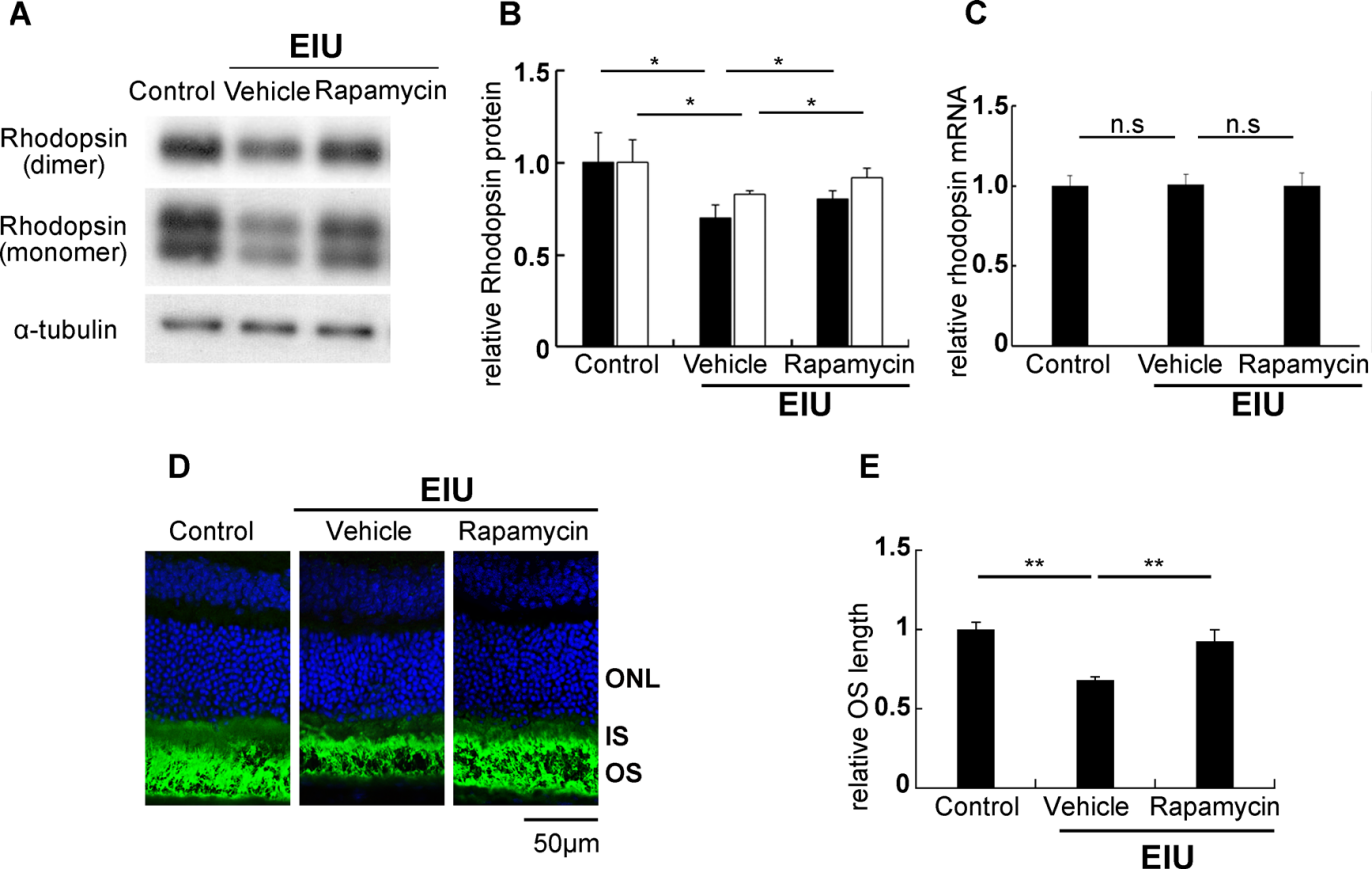
Rapamycin suppressed rhodopsin reduction and OS shortening during inflammation in a post-transcriptional manner. Immunoblot analysis showed that both the monomer and dimer forms of rhodopsin were reduced in the retina 24 hours after inflammation induced by an intraperitoneal injection of lipopolysaccharide (LPS), and that this reduction was attenuated by rapamycin treatment (A, B; monomer, black bars; dimer, white bars). Real-time PCR showed that the *rhodopsin* mRNA level was not changed in the retina during inflammation in the presence or absence of rapamycin at the same time points (C). Immunohistochemical staining for rhodopsin (green) showed that reduction of the photoreceptor outer segment (OS) length in the retina during inflammation was suppressed by rapamycin (D, E). Nuclei were counterstained with DAPI (blue). Scale bar, 50 μm. (A-C) n = 5; (D, E) n = 4. *p < 0.05.

We first analyzed the protein level of rhodopsin, a visual pigment indispensable for rod photoreceptor cells and visual function, during inflammation. Both the monomer and dimer forms of the rhodopsin protein were reduced in the retina 24 hours after LPS injection, similar to what was previously reported [19, 21, 23–26]. This reduction was significantly attenuated by rapamycin (Fig 1A and 1B). No change in the mRNA level of *rhodopsin* was observed between each treatment group at the same time point (Fig 1C). These results indicated that rapamycin suppressed the rhodopsin reduction during inflammation in a post-transcriptional manner. We also checked the photoreceptors’ OS length in the immunohistochemical stainings as rhodopsin is concentrated in the OS. We found that reduction in the OS length after LPS injection was suppressed by rapamycin (Fig 1D and 1E).

### Rapamycin suppressed visual function impairment during inflammation

**Fig 2:**
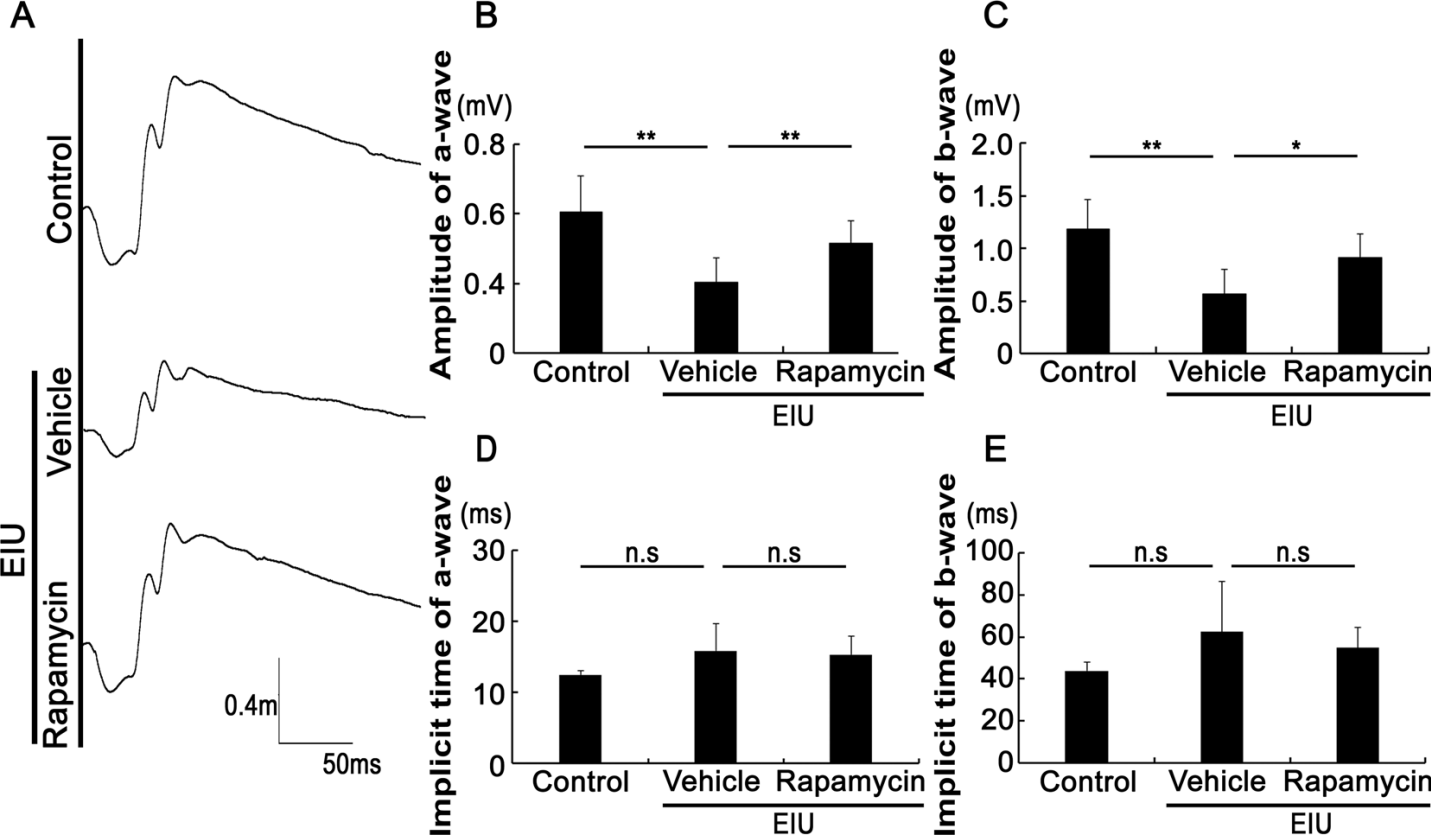
Rapamycin suppressed visual function impairment during inflammation. Representative waveforms of an individual mouse recorded by electroretinography 24 hours after the induction of inflammation by an intraperitoneal injection of lipopolysaccharide (LPS) (A). The amplitudes of both the a-wave (B) and b-wave (C) were reduced during inflammation, and were attenuated by the rapamycin treatment. The implicit times of the a-wave (D) and the b-wave (E) were not changed; n = 6. *p < 0.05, **p < 0.01.

The effect of rapamycin on visual function during inflammation was determined by electroretinography 24 hours after LPS injection (Fig 2A–2E). Consistent with the results of previous studies [19, 21, 23, 25, 26], the amplitudes of the a- and b-wave, which reflect photoreceptor function and photoreceptor-stimulated electrical activity in the inner retina, respectively, were reduced during inflammation. However, the rapamycin treatment significantly attenuated these reductions, indicating that rapamycin suppressed visual function impairment during inflammation. The implicit times of the a- and b-waves were not changed in each group.

### Rapamycin suppressed inflammatory molecules in the retina during inflammation

**Fig 3:**
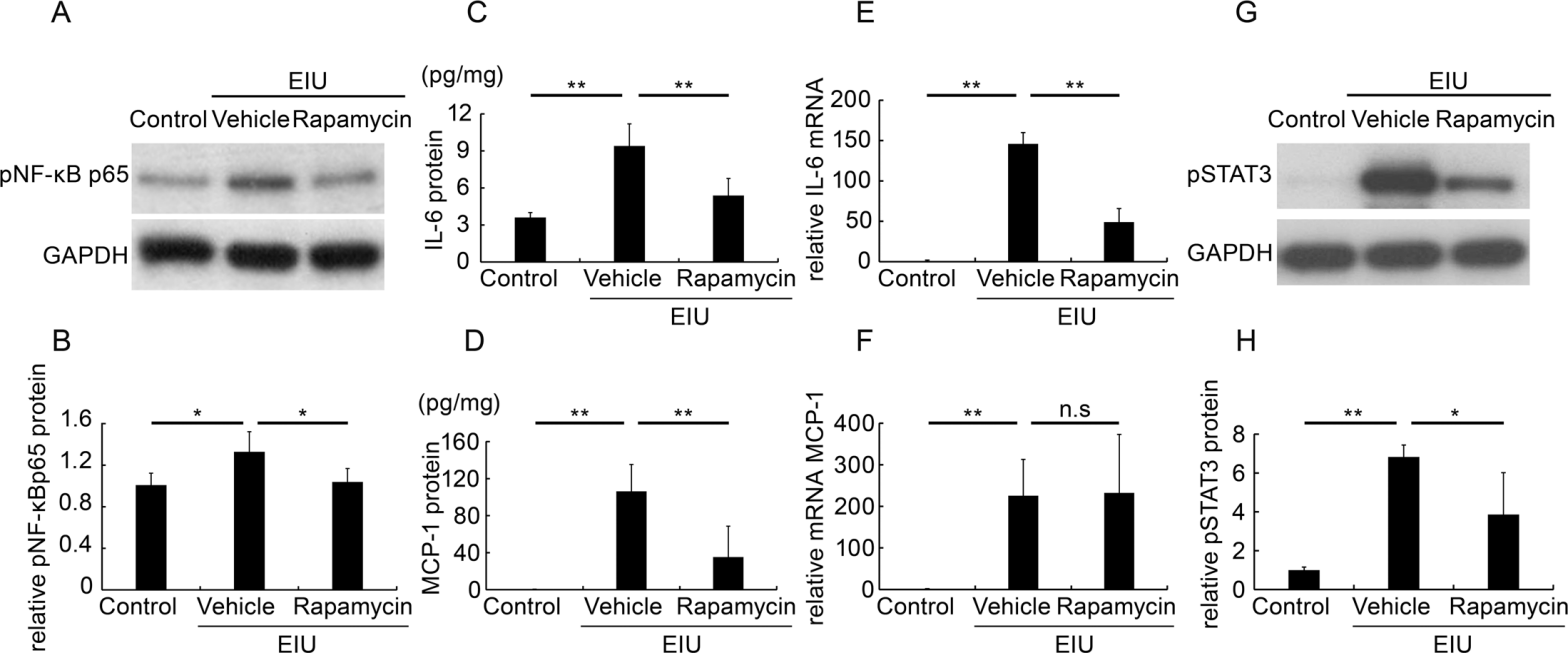
Rapamycin suppressed inflammatory molecules in the retina during inflammation. Immunoblot analysis showed that phosphorylated nuclear factor kappa-light-chain-enhancer of activated B cells p65 (pNF-κBp65) was increased in the retina 3 hours after the induction of inflammation by an intraperitoneal injection of lipopolysaccharide (LPS), and that this was attenuated by the rapamycin treatment (A, B). ELISA assays showed that the protein levels of interleukin-6 (IL-6) and monocyte chemoattractant protein-1 (MCP-1) were increased in the retina 12 hours after the induction of inflammation, and that these changes were attenuated by the rapamycin treatment (C, D). Real-time PCR showed that the mRNA levels of both *IL-6* and *MCP-1* were increased 6 hours after the induction of inflammation, and that the mRNA level of *IL-6* (E), but not that of *MCP-1* (F), was attenuated by the rapamycin treatment. Immunoblot analyses showed that the expression level of phosphorylated and activated signal transducer and activator of transcription 3 (pSTAT3) was increased at 12 hours after inflammation, and that this increase was attenuated by the rapamycin treatment (G, H); n = 5. *p < 0.05, **p < 0.01.

Next, we evaluated the levels of inflammatory proteins in the retina. Phosphorylated and activated NF-κB p65 (pNF-κB p65), which is a transcription factor upregulated during inflammation, was already increased 3 hours after LPS injection (Fig 3A and 3B). The increase was inhibited by rapamycin. Similarly, the retinal protein levels of the inflammatory cytokines IL-6 and MCP-1 were increased at 12 hours after LPS injection, and were attenuated by rapamycin (Fig 3C and 3D). Interestingly, the increase in the retinal mRNA level of *IL-6* during inflammation at 3 hours post-LPS injection was significantly suppressed by rapamycin (Fig 3E), indicating that the increase in the IL-6 protein level was, at least partly, caused by retinal cytokine production, and that this increase was suppressed by rapamycin. The retinal mRNA level of *MCP-1* was also increased during inflammation but was not affected by the rapamycin treatment (Fig 3F), suggesting a post-transcriptional regulation of MCP-1 by rapamycin. Indeed, rapamycin is known to suppress protein synthesis [29]. Taken together, these results suggested that rapamycin suppressed the production of inflammatory cytokines in the retina.

The intracellular signaling molecule known to be activated by these cytokines, STAT3, was also activated in the retina during inflammation at 12 hours after the LPS injection (Fig 3G and 3H). Similar to that of the IL-6 and MCP-1 levels, phosphorylated and activated retinal STAT3 was suppressed by rapamycin.

### Rapamycin suppressed leukocyte adhesion in the retina during inflammation

**Fig 4:**
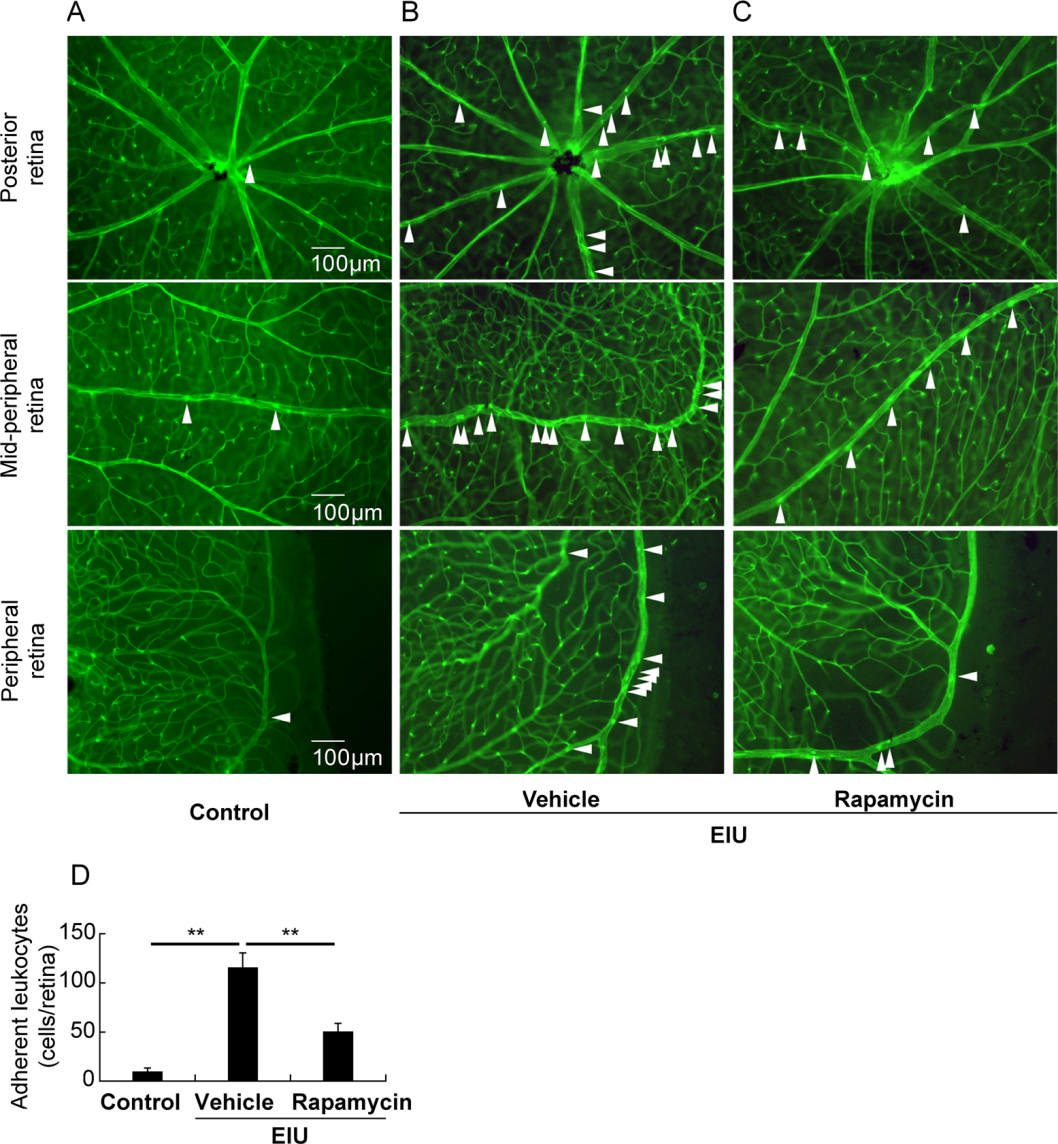
Rapamycin suppressed leukocyte adhesion in the retina during inflammation. Analysis of leukocyte adhesion in flatmount retinas showed that adherent leukocytes were minimally observed in the vehicle-treated control mice (A), and were substantially increased in the vehicle-treated EIU mice (B). However, rapamycin attenuated the number of adherent leukocytes (C). Quantification of adherent retinal leukocytes (D). Scale bar, 100 μm; n = 5. **p < 0.01.

Because the inflammatory cytokines induced in the EIU model can recruit leukocytes to the retina [20, 22, 27, 30], we also examined the leukocyte adhesion to the inner wall of retinal vessels. As expected, the number of adhesive leukocytes increased in the EIU model, and decreased after rapamycin treatment (Fig 4A–4D).

### The interaction between mTOR and NF-κB in the retina during inflammation

**Fig 5:**
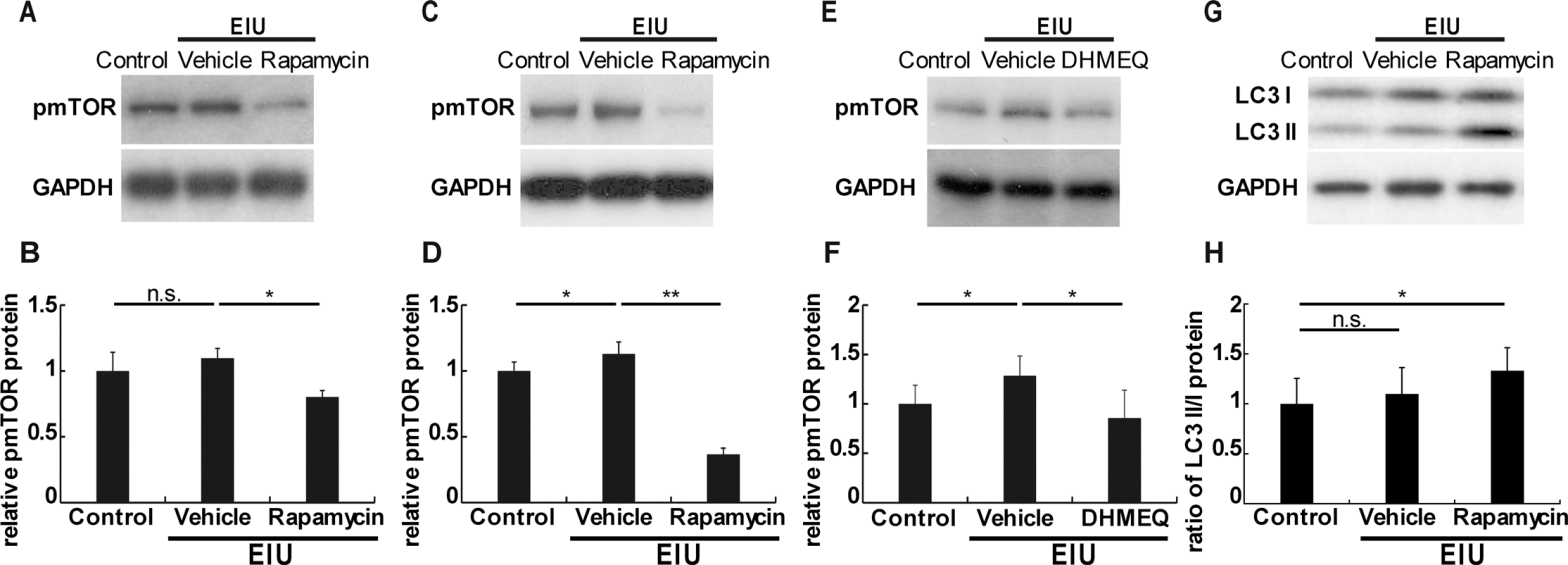
The interaction between mTOR and NF-κB in the retina during inflammation. Immunoblot analyses (A-H). The protein level of phosphorylated and activated mTOR (pmTOR) was not increased in the retina 3 hours after the induction of inflammation by an intraperitoneal lipopolysaccharide (LPS) injection, although the level of pmTOR was suppressed by the rapamycin treatment (A, B). At 12 hours after the LPS injection, the pmTOR expression was increased in the retina, which was suppressed by the rapamycin treatment (C, D). The pmTOR level in the retina at 12 hours was also suppressed by the NF-κB inhibitor, DHMEQ (E, F). The ratio of the LC3II to LC3I protein levels (an autophagy marker) in the retina was not changed after LPS injection but was increased by rapamycin at 12 hours after LPS injection (G, H); n = 5. *p < 0.05, **p < 0.01.

Because mTOR is a known target of rapamycin, we evaluated the level of phosphorylated and activated mTOR (pmTOR) in the retina during inflammation. Interestingly, while pmTOR was not elevated in the retina 3 hours after LPS injection compared with its basal expression level in the absence of inflammation, its retinal expression level was less than its basal expression level during inflammation after the rapamycin treatment (Fig 5A and 5B). However, it was increased during inflammation, 12 hours after the LPS injection, and, similarly, rapamycin significantly reduced its expression level to a level lower than that in the absence of inflammation (Fig 5C and 5D). To understand whether the increase in pmTOR at 12 hours after the LPS injection was caused by the activation of inflammatory NF-κB signaling at the onset of the inflammation, we administered DHMEQ prior to the LPS injection, and measured the pmTOR expression level at 12 hours after the LPS injection. The pmTOR expression level was reduced to that of the control group without inflammation (Fig 5E and 5F), indicating that it was increased in the retina in response to NF-κB activation during inflammation.

Because one of the known effects of rapamycin includes the activation of autophagy, which can protect neural tissue [31, 32], the LC3II to LC3I ratio (an autophagy marker) was also analyzed. Although the ratio of LC3II to LC3I was not changed in inflamed or non-inflamed retinas, it was increased and autophagy was induced by rapamycin 12 hours after LPS injection (Fig 5G and 5H). However, autophagy was not induced at 3 hours by rapamycin after LPS injection (data not shown).

## Discussion

**Fig 6:**
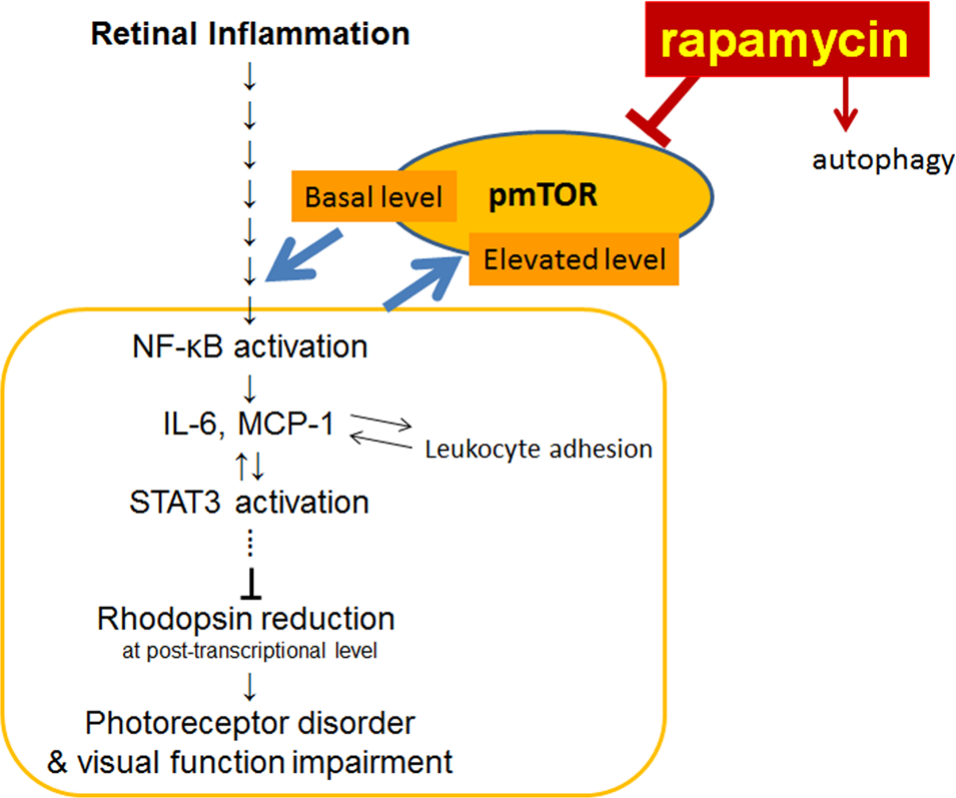
A proposed model of rapamycin’s neuroprotective effect during retinal inflammation. The basal level of activated mTOR promoted NF-κB activation during inflammation, which induced the retinal expression of the inflammatory cytokines, interleukin-6 (IL-6) and monocyte chemoattractant protein-1 (MCP-1). Activated NF-κB elevated the activated and phosphorylated mTOR (pmTOR) level during inflammation, which may have exacerbated the pathogenesis. The subsequent retinal activation of signal transducer and activator of transcription 3 (STAT3), at least partly, reduced the rhodopsin protein expression in a post-transcriptional manner and impaired rod photoreceptor function. Rapamycin also attenuated inflammatory leukocyte adhesion to the vessel walls, and increased potentially neuroprotective autophagy in the retina during inflammation.

We demonstrated that the post-transcriptional reduction in the rhodopsin protein level, OS shortening (Fig 1), and visual function impairment during inflammation were suppressed by rapamycin (Fig 2). We also showed that the NF-κB activation, IL-6 and MCP-1 levels, and the resultant STAT3 activation in the retina were suppressed by rapamycin (Fig 3). Leukocyte adhesion induced during inflammation was also suppressed by rapamycin (Fig 4). Although the activation of mTOR in the retina was induced later than that of NF-κB, the reduction in the basal level of activated mTOR by rapamycin suppressed the NF-κB activation (Fig 5). Consequently, NF-κB inhibition reduced the pmTOR level in the retina during inflammation (Fig 5), suggesting the existence of a positive feedback loop between mTOR and NF-κB during inflammation (Fig 6). Furthermore, autophagy was induced by rapamycin (Fig 5).

Retinal inflammation caused rhodopsin reduction in a post-transcriptional manner and resulted in rod photoreceptor dysfunction as measured by scotopic electroretinography. This reduction may be, at least partly, caused by excessive protein degradation through the ubiquitin proteasome system as was previously reported [21, 24]. Given that rapamycin suppresses protein synthesis [29], rapamycin’s positive effect on preserving rhodopsin in a post-transcriptional manner may be through its suppression of protein degradation rather than its promotion of protein synthesis during inflammation. Consistent with the fact that the rhodopsin level is correlated with the OS length as shown in rhodopsin knock-out mice [33], reduction in the OS length during inflammation was attenuated by rapamycin.

Rapamycin also preserved a-wave responses as shown by scotopic electroretinography, indicating that it preserved rod photorepceptor function during inflammation. Changes in the b-wave response, which represents the subsequent responses of phoroteoreceptor function, were similar to the a-wave changes, suggesting that they might have been due to the effects of the photoreceptor damage. Although rapamycin protected against secondary cone photoreceptor death in retinitis pigmentosa caused by a rod photoreceptor-specific mutation [34], our results showed that rod photoreceptors were also protected by rapamycin during innate inflammation in the retina.

NF-κB activation, which is responsible for rhodopsin reduction and visual function impairment during retinal inflammation [26], was clearly observed in the retina from an early time point after the onset of inflammation. Rapamycin suppressed this activation, suggesting that the protective effects on the rod photoreceptor cells during retinal inflammation were, at least partly, due to the suppression of retinal NF-κB activation. The suppression of NF-κB by rapamycin may be explained by the fact that mTOR activates the endogenous inhibitor of NF-κB kinase (IKK), which is upstream of NF-κB activation [35]. It is reported that mTOR controls NF-κB activity in cancer cells via the interaction with and stimulation of IKK, which is dependent on the mTOR-associated protein Raptor, while rapamycin dissociates Raptor from mTOR, thereby inhibiting IKK activation [35].

The currently ongoing clinical trial of rapamycin for the treatment of non-infectious uveitis [15] is based on the concept that rapamycin suppresses the T cell activation induced in the late inflammation phase after the host has been sensitized by the pathological antigen. However, in the present study, rapamycin’s treatment effect, including the suppression of IL-6 and MCP-1, was determined at the early inflammation phase after pathogenic induction by LPS. This suggested that T cells were less likely involved in the inflammatory pathway, and that, rather, innate inflammation in the retina might have been regulated by rapamycin. It would, therefore, be of interest to assess whether rapamycin could be used for the treatment of human innate inflammatory conditions. Inflammatory cytokines, such as IL-6 and MCP-1, are induced in multiple retinal diseases other than uveitis, e.g., diabetic retinopathy [36–39]. The results of the current study suggest that inflammation in such diseases might be treated by rapamycin.

The retinal induction of IL-6 and MCP-1 was paralleled by activation of STAT3. This is consistent with the fact that STAT3 is activated downstream of these cytokines. Since STAT3 can induce the activation of these cytokines further, a positive feedback loop enhancing the expression of IL-6 and MCP-1 and the activation of STAT3 may have been formed. Because STAT3 is responsible for rhodopsin reduction during retinal inflammation [21, 24], rapamycin’s protective effects on rhodopsin and rod photoreceptor function may be through the reduction in the IL-6 and MCP-1 expression and the resultant STAT3 activation.

Moreover, LPS-induced leukocyte adhesion to the retinal vessels was also suppressed by rapamycin. Because induction of IL-6 and MCP-1 in the retina can cause leukocyte adhesion, suppression of these cytokines may have caused leukocyte adhesion suppression. Leukocyte activation could have further induced the expression of IL-6 and MCP-1 in the local lesion [20], thereby instigating a vicious inflammatory reaction cycle, suggesting that rapamycin might also have affected this vicious inflammatory reaction cycle and efficiently have suppressed inflammation.

Strikingly, the level of pmTOR was not increased at the early inflammation phase; however, NF-κB was activated, which was suppressed by rapamycin and, consequently, mTOR activation was suppressed as well. Thus, the reduction in the basal expression level of pmTOR by rapamycin suppressed pathological NF-κB activation, indicating that the basal expression level of pmTOR was sufficient to activate NF-κB in the retina during inflammation (Fig 6). This most likely occurred because certain inflammatory triggers that required baseline expression levels of pmTOR were induced to activate NF-κB. Therefore, the reduction in the retinal pmTOR expression level from the onset of inflammation may have contributed to the prevention of NF-κB activation in response to inflammatory triggers. Thus, rapamycin might be useful during the early inflammation phase to alleviate the induction of a vicious inflammatory cycle. However, further research is warranted to investigate this potential mechanism.

mTOR activation during inflammation was also suppressed by the inhibition of NF-κB activation by DHMEQ, suggesting that a positive feedback loop also existed between mTOR and NF-κB activation during inflammation (Fig 6). In contrast to the results of the current study, previous studies showed that NF-κB could negatively regulate mTOR at the transcriptional level through receptor interacting protein 1 (RIP1), which played a key role in NF-κB activation in a glioblastoma cell line [40, 41]. However, another study showed that mTOR activation was suppressed in IKK-deficient cardiac myocytes in which NF-κB activation was suppressed, indicating that NF-κB activation was required to activate mTOR in these cardiac myocytes [42]. This was consistent with the results of the current study. The discrepancy between the studies may be explained by the difference in cell types used. Therefore, it would be of interest to further study the molecular mechanisms underlying the relationship between mTOR and NF-κB in the future.

Although autophagy was not suppressed during retinal inflammation, it was induced by rapamycin. Because autophagy is generally neuroprotective [31, 32, 43], rapamycin may have helped in increasing neuroprotective autophagy in the retina during inflammation, as abnormally modified proteins may aggregate and cause retinal dysfunction [24].

Concluding, the basal expression level of mTOR promoted NF-κB activation, which induced the retinal expression of the inflammatory cytokines IL-6 and MCP-1 during inflammation. Activated NF-κB induced mTOR activation during inflammation, which might have further exacerbated the pathologic inflammatory condition. Subsequent to the cytokine induction, STAT3 was activated in the retina, which, at least partly, reduced the rhodopsin protein expression level in a post-transcriptional manner and caused rod photoreceptor dysfunction. Inflammatory leukocyte changes could also have contributed to the exacerbation of inflammatory signaling. Our results suggest the potential use of rapamycin in neuroprotective therapies to locally suppress pmTOR and related inflammatory proteins that cause visual dysfunction during inflammation.
